# Orally Administered NSAIDs—General Characteristics and Usage in the Treatment of Temporomandibular Joint Osteoarthritis—A Narrative Review

**DOI:** 10.3390/ph14030219

**Published:** 2021-03-05

**Authors:** Marcin Derwich, Maria Mitus-Kenig, Elzbieta Pawlowska

**Affiliations:** 1 ORTODENT, Specialist Orthodontic Private Practice in Grudziadz, 86-300 Grudziadz, Poland; 2 Department of Experimental Dentistry and Prophylaxis, Medical College, Jagiellonian University in Krakow, 31-008 Krakow, Poland; maria.mitus@interia.pl; 3 Department of Orthodontics, Medical University of Lodz, 90-419 Lodz, Poland; elzbieta.pawlowska@umed.lodz.pl

**Keywords:** temporomandibular joint osteoarthritis, NSAID, treatment of temporomandibular joint disorders

## Abstract

*Background*: Temporomandibular joint osteoarthritis (TMJ OA) is a degenerative joint disease. The aim of this review was to present the general characteristics of orally administered nonsteroidal anti-inflammatory drugs (NSAIDs) and to present the efficacy of NSAIDs in the treatment of TMJ OA. *Methods*: PubMed database was analyzed with the keywords: “(temporomandibular joint) AND ((disorders) OR (osteoarthritis) AND (treatment)) AND (nonsteroidal anti-inflammatory drug)”. After screening of 180 results, 6 studies have been included in this narrative review. *Results and Conclusions*: Nonsteroidal anti-inflammatory drugs are one of the most commonly used drugs for alleviation of pain localized in the orofacial area. The majority of articles predominantly examined and described diclofenac sodium in the treatment of pain in the course of TMJ OA. Because of the limited number of randomized studies evaluating the efficacy of NSAIDs in the treatment of TMJ OA, as well as high heterogeneity of published researches, it seems impossible to draw up unequivocal recommendations for the usage of NSAIDs in the treatment of TMJ OA. However, it is highly recommended to use the lowest effective dose of NSAIDs for the shortest possible time. Moreover, in patients with increased risk of gastrointestinal complications, supplementary gastroprotective agents should be prescribed.

## 1. Temporomandibular Joint Osteoarthritis (TMJ OA)

**Figure 1:**
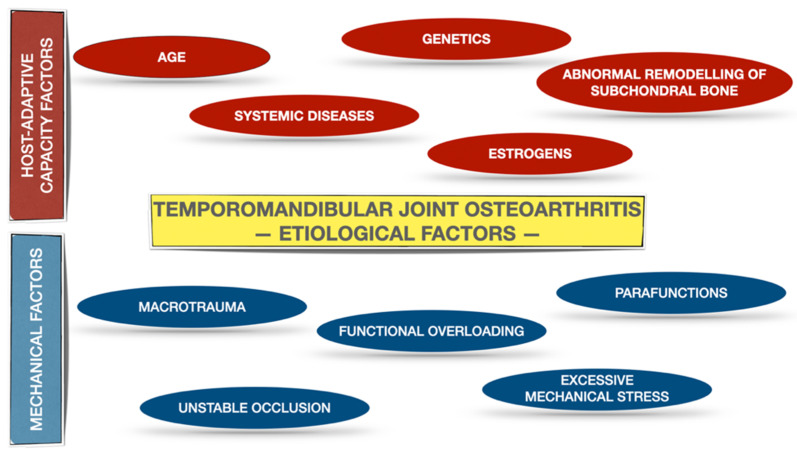
Etiological factors for TMJ OA on the basis of the literature [3,4]. The etiological factors have been allocated into two subgroups: host-adaptive capacity factors and mechanical factors.

Osteoarthritis is found to be the most widespread joint disease, which refers to the entire joint, including not only the articular cartilage and subchondral bone, but also joint capsule, synovial membrane, ligaments and even adjacent muscles [1,2,3]. TMJ OA is a low-inflammatory arthritic disorder [3,4]. According to the Diagnostic Criteria for Temporomandibular Disorders (DC/TMD) for Clinical and Research Applications, TMJ OA was subclassified as degenerative joint disease (DJD), ICD-10 M19.91 [5]. The prevalence of DJD in TMJ is higher in the group of patients who suffer from systemic rheumatic diseases (on juvenile idiopathic arthritis patients the prevalence of DJD ranged from 40.42% to 93.33%, whereas on rheumatoid arthritis patients the prevalence of DJD ranged from 45.00% to 92.85%) compared to the patients with TMJ disorders (TMD) with no systemic arthritic diseases diagnosed (the prevalence of DJD ranged from 18.01% to 84.74%) [6]. The etiology of DJD is multifactorial and encompasses both the host-adaptive capacity factor, as well as mechanical factors, including TMJ overloading [3,4]. Figure 1 presents etiological factors leading to the development of the TMJ OA on the basis of the literature [3,4].

**Figure 2:**
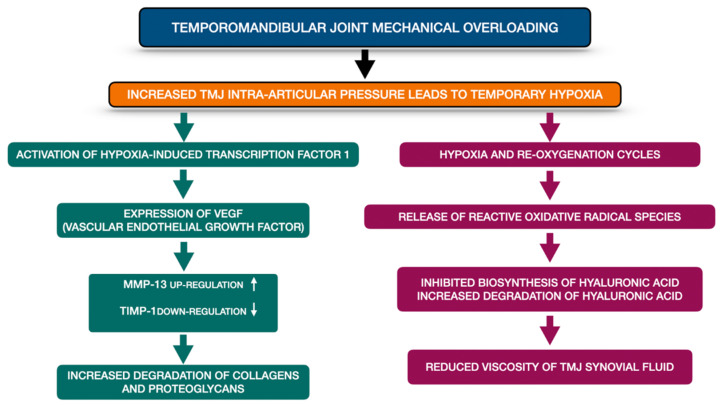
Schematic changes occurring within the overloaded TMJ, leading to extracellular matrix remodeling as well as to increased friction within the TMJ on the basis of the literature [4]. MMP-13—metalloproteinase 13; TIMP-1—tissue inhibitor of matrix metalloproteinases-1, TMJ—temporomandibular joint, VEGF—vascular endothelial growth factor.

TMJ mechanical overloading is one of the crucial TMJ OA etiological factors. It increases the TMJ intra-articular pressure and consequently causes temporary hypoxia. Presence of hypoxia within the TMJ induces activation of hypoxia-induced transcription factor-1, which afterwards stimulates expression of vascular endothelial growth factor (VEGF). VEGF provokes chondrocytes to produce metalloproteinases (MMP-13 up-regulation) and at the same time to reduce the expression of tissue inhibitor of matrix metalloproteinases (TIMP-1 down regulation). Finally, the extracellular matrix becomes remodeled by increased degradation of collagens and proteoglycans. Apart from this, temporary hypoxia may lead to hyaluronic acid degradation. Repetitive cycles of temporary hypoxia and re-oxygenation contribute to release of reactive oxidative radical species. Not only do the reactive oxidative radical species inhibit the biosynthesis of hyaluronic acid, but also, they increase degradation of already produced hyaluronic acid. The abovementioned changes reduce the viscosity of TMJ synovial fluid. Figure 2 presents schematic changes occurring within the overloaded TMJ, leading to extracellular matrix remodeling as well as to increased friction within the TMJ on the basis of the literature [4].

DJD can be diagnosed when the patient reports any type of the TMJ noises either within 30 days before or during the examination and the crepitus is present during at least one of the mandibular movements while the TMJ examination is taking place. The DJD diagnosis based only on the clinical examination and case history is characterized by low sensitivity (0.55) and low specificity (0.61) [5]. Therefore, TMJ computed tomography or cone-beam computed tomography imaging is recommended to confirm the DJD diagnosis [5,7,8,9]. Subcortical cysts, surface erosion, generalized sclerosis and the presence of osteophytes are typical radiological signs of TMJ OA [5,9]. Although subcortical sclerosis and articular surface flattening were also reported as osteoarthritic bony changes [8], some of the authors classify them as indeterminate for OA, because of the fact that they may represent remodeling, normal variations and changes associated with age [5,9]. The term TMJ OA is used when apart from the presence of the abovementioned DJD diagnostic criteria, patients report joint pain [10]. There are several other symptoms which may occur in the course of TMJ OA, including: loss of joint function, TMJ ankylosis and apertognathia that results from the loss of posterior vertical dimension of the mandible [4].

## 2. Treatment of TMJ OA

Treatment of TMJ OA requires an interdisciplinary approach [11,12]. There are three major aims of TMJ OA treatment: symptom relief including pain control, inactivation of the disease and finally retrieval of normal joint function [3]. Different methods of TMJ OA treatment have been grouped into several categories from the least to the most invasive procedures [11,13]. The abovementioned categories include: conservative treatment, including physiotherapy, occlusal splint therapy and pharmacotherapy; less invasive surgical procedures, including injections into the joint and arthrocentesis; and finally, invasive surgical procedures (arthroscopy and open joint surgery) [11,13].

**Figure 3:**
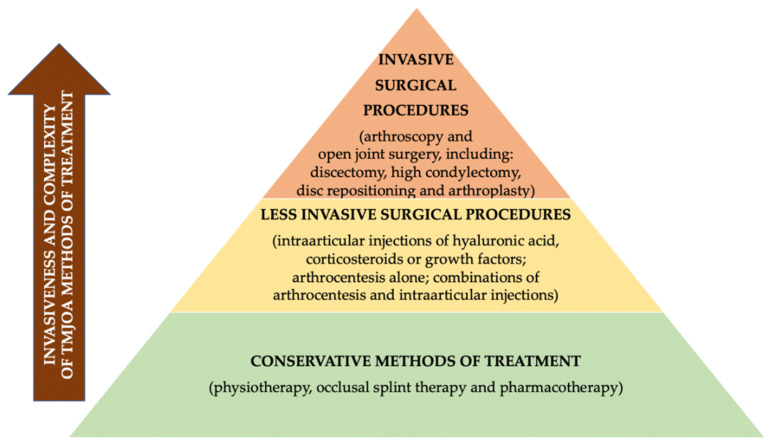
Hierarchy pyramid presenting different methods of TMJ OA treatment on the basis of the literature [11,13].

Figure 3 presents the hierarchy pyramid presenting different methods of TMJ OA treatment on the basis of the literature [11,13].

The aim of this narrative review was to present the general characteristics of orally administered nonsteroidal anti-inflammatory drugs (NSAIDs) and to present the efficacy of NSAIDs in the treatment of TMJ OA.

## 3. Biosynthesis of Eicosanoids

Eicosanoids are molecules biosynthesized from arachidonic acid. Arachidonic acid is liberated from membrane phospholipids by the enzyme phospholipase A2 [14]. There are several subcategories of eicosanoids, including among others: leukotrienes, lipoxins and prostanoids [15]. Cyclooxygenases 1 and 2 (COX-1 and COX-2), also known as prostaglandin-endoperoxide synthases 1 and 2 (PTGS1 and PTGS2), play significant roles in prostanoids biosynthesis due to the fact that they transform arachidonic acid to prostaglandin G2 (PGG2), which is subsequently converted into prostaglandin H2 (PGH2) [15,16]. Prostanoid synthase enzymes form prostaglandin E2 (PGE2), prostaglandin F2α (PGF2α), prostaglandin D2 (PG2), prostacyclin (PGI2) and thromboxane A2 (TxA2) from prostaglandin H2 (PGH2) [15,17]. Prostaglandins, especially PGE2, contribute to gastric cytoprotection mostly by the reduction of the amount of secreted hydrochloric acid as well as by stimulation of mucus secretion [18]. Moreover, prostaglandins were found to increase the neuronal excitability, leading to a pro-nociceptive effect [19,20]. Prostacyclin appears in vascular endothelial cells and is responsible for vasodilatation and platelet antiaggregatory effect. Furthermore, prostacyclin also presents pro-nociceptive action [19]. Thromboxane A2, localized in platelets, leads to vasoconstriction as well as stimulates platelet aggregation [19].

## 4. NSAIDs Inhibit the Activity of Cyclooxygenases

Non-steroidal anti-inflammatory drugs (NSAIDs) are one of the most commonly used analgesics for the treatment of pain in the orofacial area [21,22]. NSAIDs may be classified on the basis of their chemical structure into five subgroups: salicylic acid derivatives (i.e., sodium salicylate, acetylsalicylic acid), aryl and heteroaryl acetic acid derivatives (i.e., ibuprofen and naproxen), indole and indene acetic acid derivatives (i.e., indomethacin, etodolac), anthranilates (i.e., diclofenac, mefenamic acid) and enolic acid derivatives (i.e., piroxicam, meloxicam) [23]. NSAIDs present different plasma half-life and therefore should be subdivided into two subgroups: short-acting NSAIDs with plasma half-life up to 6 h (i.e., diclofenac, aspirin, ibuprofen) and long-acting NSAIDs with plasma half-life over 10 h (i.e., celecoxib, naproxen) [23].

NSAIDs inhibit the activity of cyclooxygenases, enzymes localized in the endoplasmic reticulum [16,21,22,24]. There have been described three isoforms of cyclooxygenase: COX-1, COX-2 and, localized mainly in the central nervous system, COX-3 [16,24,25,26,27].

COX-1 is a constitutive isoenzyme, which is permanently present in many tissues and takes part in several physiological processes. Unlike COX-1, COX-2 is expressed constitutively only in a few organs, including the brain, kidneys and uterus. COX-2 is primarily an inducible isoform of cyclooxygenase [24,27]. Both isoforms are responsible for the same biochemical reactions, but they differ in the morphology of the active sites. The COX-1 active site has been found to be smaller than the COX-2 active site [16].

Expression of COX-2 is related to the presence of proinflammatory cytokines and growth factors [24,27]. It has been proven that osteoarthritic cartilage slices coming from patients diagnosed with severe knee OA presented 50-fold greater production of prostaglandin E2 (PGE2) comparing to the spontaneous production of PGE2 by normal cartilage. Osteoarthritic cartilage presented a coincidence between superinduction of PGE2 production and upregulation of COX-2 [28]. Proinflammatory cytokine, IL-1 beta, was found to induce high expression of COX-2 and subsequently increased production of PGE2 in osteoarthritic tissues. Moreover, it has been shown that PGE2 coming from COX-2 modulates cartilage proteoglycan degradation in the course of OA [29]. In addition to this, it has been discovered that expression of COX-2 is directly associated with synovitis and joint pain in patients with internal derangement or TMJ OA [30]. Therefore, COX-2 inhibition is needed to reduce pain and inflammation in the area of TMJ. Celecoxib, one of the selective COX-2 inhibitors, has been found to present a positive, protective effect on mandibular condylar chondrocytes that were under cyclic tensile strain. Celecoxib reduces degradation and increases biosynthesis of mandibular condylar chondrocytes extracellular matrix [31].

Inhibition of constitutive forms of cyclooxygenases leads to side effects of NSAIDs, whereas inhibition of inducible forms of cyclooxygenase 2 (COX-2) produces therapeutic effects of NSAIDs [15].

## 5. COX-1 and COX-2 Selectivity of NSAIDs

The majority of NSAIDs, including acetylsalicylic acid (aspirin), ibuprofen, naproxen and ketoprofen, are nonselective inhibitors of cyclooxygenase, which means they block both isoforms: COX-1 and COX-2. Cryer and Feldman [32] assessed the concentration of nonselective NSAIDs that inhibited 50% of cyclooxygenase activity (IC50). According to their study, the highest inhibitory potency for COX-1 in blood was presented by ketoprofen, indomethacin, diclofenac, ketorolac and flurbiprofen, whereas the highest inhibitory potency for COX-2 in blood was presented by diclofenac, valeryl salicylate, dexamethasone, mefenamic acid and nimesulide. Diclofenac appeared to be the most potent and selective COX-2 inhibitor. Although its selectivity was 20 times higher for COX-2 than COX-1, diclofenac was still an effective inhibitor of COX-1 [32]. Therefore, diclofenac presents a very low risk of NSAID-associated gastrointestinal (GI) incidents, but at the same time it highly increases the risk of NSAID-associated cardiovascular events [33]. This is thoroughly explained later on. 

Selective COX-2 inhibitors (celecoxib, etoricoxib) preferably block COX-2 isoform. When used alone, they present a lower risk of GI complications, whereas if combined with low-dose aspirin, the risk of upper GI ulcer bleeding is similar to nonselective COX inhibitors [34]. 

## 6. Adverse Effects of NSAIDs

There are several possible adverse effects of taking NSAIDs, which may occur at any time throughout the whole treatment period. The most commonly described are: gastrointestinal and cardiovascular complications. There are several other adverse effects of NSAIDs, including: hepatic complications, impaired renal function, clotting problems, respiratory disorders (aspirin-exacerbated respiratory disease), as well as prolonged pregnancy or labor [21,35,36,37,38].

GI complications may occur in up to 60% of patients who administer long-term NSAIDs [39,40,41,42,43,44]. There are two mechanisms in which NSAIDs induce GI injury: the topical and the systemic ones. The majority of NSAIDs present acidic properties. Therefore, the topical mechanism refers to the direct injury to gastric mucosa caused by NSAIDs, whereas the main reason explaining the presence of systemic adverse effects of NSAIDs is the fact that nonselective NSAIDs inhibit constitutive COX forms and therefore lead to the decreased biosynthesis of prostaglandins, affecting both hydrochloric acid and mucus secretion [21,35,36,45,46]. Sostres et al. [36] subcategorized different NSAIDs adverse effects related to upper GI tract into four groups: symptoms like dyspepsia, nausea, vomiting, abdominal pain and heartburn; NSAIDs related gastroduodenal injury with unclear clinical significance; symptomatic ulcers and GI complications (GI bleeding, ulcer perforation and obstruction); mortality. 

There are several risk factors for GI complications in patients who take NSAIDs. These risk factors include (from the highest risk): history of complicated ulcers; simultaneous use of anticoagulants; multiple NSAID use, including low-dose aspirin; history of uncomplicated ulcer; high doses of NSAID (or use of piroxicam or ketorolac); age above 60 years; severe illness; Helicobacter pylori infection; concomitant use of corticosteroids [36,47,48]. 

Both the nonselective COX inhibitors (apart from aspirin) and selective COX-2 inhibitors are associated with the increased risk of acute cardiovascular events [49,50]. There have been discussed two potential mechanisms responsible for coronary events, namely: COX-1/COX-2 selectivity and renal effects in the course of the long-term renal COX-2 inhibition, leading to reduced sodium excretion, water retention and finally increased blood pressure [51]. Both of the abovementioned mechanisms are dose-dependent and duration-dependent [51]. Although, the hypothesis regarding the relationship between the risk of myocardial infarction and COX-2 selectivity has not been confirmed, there are two NSAIDs: diclofenac (nonselective COX inhibitor) and rofecoxib (selective COX-2 inhibitor), which used long-term and in high doses, have been found to be associated with the increased risk of myocardial infarction [51]. Coxib and traditional NSAID Trialists’ (CNT) Collaboration [52] found that both diclofenac and coxibs comparably increased vascular risk. Moreover, they also noticed that ibuprofen significantly increased major coronary (not vascular) events, whereas, naproxen appeared to be the safest NSAID, because it did not increase vascular or coronary events [52].

NSAIDs were also found to induce reactive oxygen species also in cells related to the cardiovascular system. There are several sources of reactive oxygen species in the cardiovascular system, including among others: mitochondria, xanthine oxidoreductases and nitric oxide synthases. Additional reactive oxygen species, induced by NSAIDs, may initiate oxidative stress, leading to cell apoptosis and finally to cardiotoxicity [53].

There are a few high-risk factors for NSAID-associated cardiovascular incidents, including patients with history of acute coronary syndrome, patients with percutaneous/ surgical coronary revascularization, patients diagnosed with stable angina and angiographic evidence of significant coronary artery stenosis, patients with a history of stroke, patients with documented significant carotid artery stenosis, as well as patients with congestive heart failure [33].

## 7. Prevention of Adverse Effects of NSAIDs

TMJ OA therapy with NSAIDs lasts most often from two to four weeks [21]. Sometimes, if needed, NSAIDs may be administered for even longer. Long-term administration of NSAIDs increases the risk of previously listed adverse effects. Therefore, it is highly recommended to use the lowest effective dose of NSAIDs for the shortest possible time to diminish the risk of the previously described side effects [33,34,35,36,49,50,54].

To reduce the risk of NSAID-associated GI adverse effects, supplementary gastroprotective agents should be prescribed [33,37,44,55]. There are several drugs which serve as gastroprotective agents, including: proton pump inhibitors (PPIs), high doses H2-receptor antagonists and misoprostol [36,44]. Proton pump inhibitors stop gastric acid secretion by inhibition of hydrogen–potassium pumps [56,57,58]. Therefore, they are very effective in reducing upper GI complications, especially ulcers and mucosal injuries, induced by NSAIDs administration [56,57]. PPIs have been found to be more effective than high doses of H2-receptor antagonists or misoprostol for healing ulcers related to NSAIDs [56,57]. PPIs do not protect from lower GI complications related to NSAIDs. Moreover, PPIs were found to induce dysbiosis of the small intestinal bacterial flora, which is considered as a small intestinal bacterial overgrowth (SIBO). This adverse effect may exacerbate the small intestinal injuries caused by NSAIDs [58,59]. Moreover, long-term usage of PPIs may also be associated with several other potential adverse effects, including: infections, micronutrient deficiencies, bone fracture, kidney disease and dementia. However, it should be noted that the overall quality of evidence regarding the potential PPI-associated adverse effects is either low or very low [56,60]. It is emphasized that, especially for patients with a high risk of GI and cardiovascular complications, communication between different specialists, including gastroenterologists, cardiologists and primary care physicians, is mandatory [61].

Ho et al. [33] presented the treatment algorithm for choice of NSAIDs to reduce the risk that potential NSAIDs adverse effects may occur. The authors recommended to ensure normal renal function (eGFR > 60 mL/min) and to assess possible risk factors for cardiovascular and GI complications. Patients with low GI risk and high cardiovascular risk should be prescribed either low-dose celecoxib (200 mg/day) or naproxen with proton pump inhibitor, whereas patients with low GI risk and low cardiovascular risk are recommended to administer either celecoxib or any nonspecific NSAID with proton pump inhibitor. Patients with high GI risk and high cardiovascular risk should receive low-dose celecoxib (200 mg/day) with proton pump inhibitor or opioids ought to be considered. Finally, patients with high GI risk and low cardiovascular risk should be prescribed celecoxib with proton pump inhibitor [33].

## 8. Recent Advances in NSAIDs Development

Recent researches related to NSAIDs are focused on the production of at least equally efficient anti-inflammatory drugs with a significantly decreased number of side effects. Rao et al. [62] discussed six novel groups of small molecule drugs, including: NO–NSAIDs, selective COX-2, dual COX/LOX, lipoprotein-PLA2 (Lp- PLA2), mPGES-1 and TNF- α inhibitors.

Nitric oxide–NSAIDs (NO–NSAIDs) are hybrid NSAIDs, which contain NO-donor groups. The major advantages related to the presence of nitric oxide are: vasodilatation, inhibition of platelet aggregation, as well as GI mucosal healing. Therefore, NO–NSAIDs could reduce both GI and cardiovascular side effects related to traditional NSAIDs [63]. NO–NSAIDs present different interactions with phospholipid bilayers compared to traditional NSAIDs, which may have clinically significant implications on gastric mucosa. Pereira-Leite et al. [64] presented the study comparing interactions of NO-indomethacin and indomethacin with phospholipid bilayers. The authors found that NO-indomethacin, compared to indomethacin, led to more pronounced changes in the biophysical properties of phospholipid bilayers. Moreover, Aisa et al. [65] noticed that NO-derivatives of aspirin and naproxen not only did not change osteoblast proliferation and differentiation, but also reduced the activity of plasminogen activator, metalloproteinases and cathepsin B. The authors concluded that NO–NSAIDs present a safer impact on metabolism of osteoblasts compared to celecoxib [65].

Another example of so-called safer anti-inflammatory drugs are dual COX-2/5-LOX inhibitors. These drugs inhibit not only cyclooxygenase, but also lipoxygenase pathways. Lipoxygenases transform arachidonic acid to leukotrienes, which take part in both inflammatory processes and tumor development. The main role of COX-2/5-LOX inhibitors was to eliminate all of the inflammatory mediators coming from the arachidonic acid pathways [66,67,68,69,70]. 

Microsomal prostaglandin E synthase-1 (mPGES-1) inhibitors selectively reduce the biosynthesis of prostaglandin E2 (PGE2). Because of undisturbed biosynthesis of other prostanoids, this group of drugs was expected to present a decreased number of side effects. In fact, although mPGES-1 inhibitors appeared to be cardioprotective, they still have not been approved for clinical practice. The major problem is associated with interspecies differences in the morphology of the mPGES-1 between humans and rodents [71,72].

Although, there have been performed several researches regarding the development of the abovementioned new x-inflammatory drugs, still little is known about pharmacological actions of novel drugs in humans and further studies are needed.

## 9. NSAIDs in the Treatment of TMJ OA

Unfortunately, the number of studies discussing the efficacy of NSAIDs in the treatment of the TMJ disorders, including TMJ OA, is very limited. 

Mejersjö et al. [73] presented a randomized, single-blind study, which aimed to compare the efficacy of occlusal splint therapy (15 patients) and therapy with diclofenac sodium (14 patients) in the treatment of the TMJ OA. Voltaren (diclofenac sodium) was administered three times per day at a dose of 50 mg. The dosage was limited to 50 mg every 12 h, when the TMD symptoms were reduced. Both groups had been treated for three months. Although diclofenac led to more rapid improvement of TMD symptoms, the obtained results showed no statistically significant differences between the two groups. However, it must be emphasized that diclofenac administered at a dose of 150 mg per day for at least 90 days was found to be associated with increased risk of myocardial infarction or cardiovascular death [51]. Kurita Varoli et al. [74] presented similar observations, but used different methodology. The authors assessed the group of patients who had been suffering from chronic pain of masticatory muscles due to the TMD. All of the participants received occlusal splints and three different drug therapies: NSAID (50 mg sodium diclofenac twice a day for 10 days), panacea (300 mg acetaminophen + 50 mg sodium diclofenac + 125 mg carisoprodol + 30 mg caffeine twice a day for 10 days) and placebo (twice a day for 10 days). There were 11-day washout periods between different drug therapies. During washout periods patients did not wear occlusal splints or take any medicaments. According to the obtained results, occlusal splints with either NSAID or panacea reduced pain during the third day of treatment, whereas occlusal splints with placebo reduced pain during the eighth day of treatment. There were no differences between the groups regarding the obtained analgesic effect after 10-day treatment periods. These results clearly presented the positive aspect of adjuvant sodium diclofenac administration to occlusal splint therapy on earlier pain reduction. Song et al. [75] found that therapy with occlusal stabilization splints as well as administration of NSAIDs significantly improved TMJ OA. The pharmacotherapy with diclofenac sodium lasted 61.91 ± 42.15 days (the dosage was not presented). Because of the fact that several patients received simultaneously different methods of treatment, it seems impossible to distinguish the exact impact of NSAIDs vs. splint therapy on TMJ OA therapy. The authors recommended to combine both methods of treatment: occlusal splint therapy and administration of NSAIDs to reduce the TMJ mechanical overloading, as well as to remove chemically inflammatory mediators from the TMJ. This summary agrees with the previously described conclusions by Kurita Varoli et al. [74]. Dalewski et al. [76] compared the effectiveness of occlusal splints alone, occlusal splints combined with dry needling and occlusal splints combined with NSAID (nimesulide 100 mg every 12 h for 14 days) in the treatment of unilateral pain in the area of TMJ. Although the observation time was longer (three weeks) compared to the research by Kurita Varoli et al. [74] (10 days per each drug protocol), the authors noticed that combination of occlusal splint with nimesulide led to significantly better pain relief compared to the remaining two groups. However, it must be noted that the occlusal splints were worn by the patients at night time only. If the occlusal splints had been worn by the patients all day and night, the results could have been different. 

The only research which analyzed different methods of diclofenac sodium administration was presented by Di Rienzo Businco et al. [77]. The authors observed that all of the patients with TMJ pain who had received either 50 mg of diclofenac sodium every 12 h for 14 days administered orally or 16 mg/mL topical diclofenac (diclofenac topical solution, 10 drops 4 times a day for 14 days) presented pain relief. The results obtained by the authors indicate that lower doses of diclofenac sodium administered orally for shorter periods of time give effective pain relief. Moreover, the authors proved that there was no difference regarding the efficacy of both oral administration and multidose topical application of diclofenac sodium. Topical application eliminates the possible risk of adverse systemic effects, which may occur when diclofenac sodium is administered orally. 

Ta et al. [78] evaluated the efficacy of celecoxib, naproxen and placebo in patients with painful TMJs secondary to disc displacement with reduction. Patients received one of the below mentioned: celecoxib 100 mg twice a day, naproxen 500 mg twice a day, or placebo for six weeks. Naproxen appeared to be most effective in TMJ pain reduction, whereas celecoxib was just slightly better than placebo. Naproxen reduced significantly TMJ pain intensity within three weeks of treatment. According to the authors, to achieve the highest efficacy of TMJ pain reduction, both COX isoforms—COX-1 and COX-2—need to be inhibited.

Despite the fact that several studies presented positive effects of NSAIDs on TMJ pain relief, it is nearly impossible to assess the direct effectiveness of NSAIDs, mostly because of researches heterogeneity [79], including multimodal ways of treatment. Moreover, the presented results should not be generalized due to the very limited number of studies [80].

Table 1 presents exemplary orally administered NSAIDs available in Poland which can be used for the treatment of TMJ OA on the basis of Pharmindex [81].

Table 2 presents the effectiveness of orally administered NSAIDs in the treatment of TMJ OA on the basis of the literature [73,74,75,76,77,78].

## 10. Materials and Methods

### 10.1. Clinical Question

What is the efficacy of orally administered pharmaceuticals: nonsteroidal anti-inflammatory drugs (NSAIDs) in the treatment of temporomandibular joint osteoarthritis (TMJ OA) on the basis of the literature?

### 10.2. The PICO Approach

We used the PICO approach to properly develop literature search strategies for this narrative review: 

*Population*: adult patients (aged: 18 years old or more) who were diagnosed with TMJ OA.

*Intervention*: pharmacological treatment of TMJ OA with orally administered NSAIDs.

*Comparison*: different pharmacological treatment, occlusal splints, placebo, no treatment.

*Outcome*: decreased pain in the TMJ area and increased maximum mouth opening. 

### 10.3. Search Strategy

PubMed database was analyzed with the keywords: (temporomandibular joint) AND ((disorders) OR (osteoarthritis) AND (treatment)) AND (nonsteroidal anti-inflammatory drug). After screening of 180 results, six studies have been included in this narrative review. Primarily, we were looking for randomized clinical trials (RCTs). However, we decided to include also one case-control study because of the interesting results.

**Figure 4:**
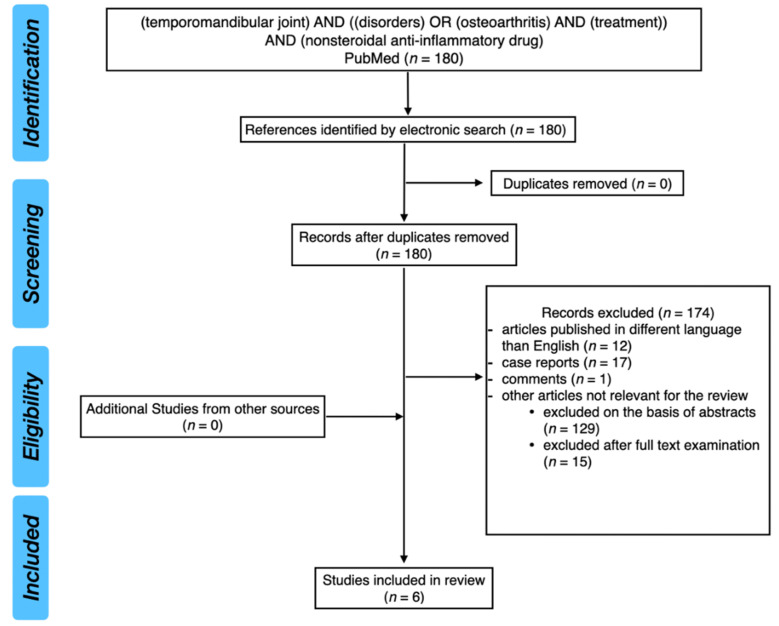
PRISMA flow diagram for review of the literature.

Figure 4 presents PRISMA flow diagram for review of the literature.

## 11. Conclusions

NSAIDs are one of the most commonly used drugs for alleviation of pain localized in the orofacial area. Unfortunately, because of the limited number of randomized studies evaluating the efficacy of NSAIDs in the treatment of TMJ OA, as well as high heterogeneity of published researches, it seems impossible to draw up unequivocal recommendations for TMJ OA treatment. However, it is highly recommended to use the lowest effective dose of NSAIDs for the shortest possible time to reduce the risk of both GI and cardiovascular complications. Moreover, in patients with the increased risk of GI complications, supplementary gastroprotective agents, including PPIs, should be prescribed.

The majority of articles predominantly examined and described diclofenac sodium in the treatment of pain in the course of TMJ OA. Diclofenac is a nonselective inhibitor of cyclooxygenase and is characterized by the lowest (among different NSAIDs) half maximal inhibitory concentration (IC50) for COX-2 and one of the lowest IC50 for COX-1. Therefore, the daily dosage of 150 mg is enough to reduce pain efficiently. Diclofenac must not be prescribed for children and adolescents under 18 years of age. Apart from this, it must be emphasized that long-term (three months) administration of diclofenac in high doses (150 mg per day) significantly increases the risk of myocardial infarction. A combination of both splint therapy and oral diclofenac administration may be recommended to achieve earlier pain relief. This can be very beneficial especially for patients who suffer from severe pain in the TMJ area in the course of TMJ OA. In such cases, diclofenac should be prescribed in the dosage of 150 mg per day for up to 14 days. 

Concluding, further long-term, randomized, double-blind trials ought to be performed to prepare general recommendations for the pharmacological supplementary therapy of the TMJ OA.

## Figures and Tables

**Table 1:** Exemplary orally administered NSAIDs available in Poland which can be used for the treatment of TMJ OA on the basis of Pharmindex [81].

Exemplary Medicinal Product	Oral Dosage (Only for Adults)	Maximum Daily Dose	Additional Information
Ibuprofen	400 mg every 6–8 h	2400 mg	Single dose more than 400 mg does not cause stronger analgesic effect.
Ketoprofen	100 mg every 12 h	200 mg	Tablets should be taken with food and at least 100 mL of milk or water.
Naproxen	250–500 mg every 12 h	1250 mg	In case of acute OA, it is recommended to increase the oral dosage from 750 mg to 1000 mg.
Diclofenac	75 mg once daily or 75 mg every 12 h	150 mg	The extended-release tablets should be taken once a dayin the dosage of 100 mg.
Celecoxib	200 mg once daily or 100 mg every 12 h	400 mg	Capsules should be taken with 200 mL of water; celecoxib may be taken with or without food; in case of acute pain patient may be prescribed initial dose of 400 mg.
Meloxicam	7.5 mg once daily	15 mg	Used for short-term treatment in case of OA exacerbation; patients aged 65 or more should not take more than 7.5 mg of Meloxicam per day.
Nimesulide	100 mg every 12 h	200 mg	Medicament should be taken after meal; the maximum duration of a treatment cycle with nimesulide is 15 days.

**Table 2:** Effectiveness of orally administered NSAIDs used in the treatment of the TMJ OA on the basis of the literature [73,74,75,76,77,78].

Reference	Study Design	Participants and Intervention	Endpoint and Results
Mejersjö et al. (2008) [73]	Randomized, single-blind study	29 patients (27 women, 2 men, aged 39–76 years): - diclofenac sodium 50 mg 3 x/day (14 patients) - splint therapy (15 patients)—there was no information on how the splints had been worn.	*Endpoint*: three months (one year follow-up) Both methods led to significant reduction of symptoms of TMJ OA within three months, but the Diclofenac group presented more rapid improvement.
Kurita Varoli et al. (2015) [74]	Randomized, triple-blind clinical trial; crossover methodology	18 patients (no precise information regarding sex, aged 35–70). All patients submitted all treatments in different moments): - splint therapy (worn during all the 10 days within treatment periods during different drug therapies) - NSAID 2 x/day for 10 days (50 mg sodium diclofenac) - 11-day washout interval (no drugs and no splint) - panacea 2 x/day for 10 days (300 mg acetaminophen + 50 mg sodium diclofenac + 125 mg carisoprodol + 30 mg caffeine) - 11-day washout interval (no drugs and no splint) - placebo 2 x/day for 10 days.	*Endpoint*: 10 days (each treatment modality) All therapies were effective for pain relief after 10 days of therapy. Sodium diclofenac used in combination with occlusal splint (both NSAID and panacea groups) led to earlier pain relief (third day) compared to placebo (eighth day).
Song et al. (2020) [75]	Case-control study	89 patients (76 women and 13 men, 152 joints), mean age of 33.17 ± 17.65 years. Treatment modalities applied to the patients: - splint therapy (72 patients) - intra-articular injections using hyaluronic acid and triamcinolone (21 patients) - diclofenac sodium (45 patients; no information about the dosage).	*Mean follow-up period*: 376.61 ± 94.49 days *Mean occlusal splint therapy period:* 524.49 ± 258.57 days *Mean duration of NSAID prescription*: 61.91 ± 42.15 days Occlusal stabilization splint therapy and NSAIDs had a significant influence on TMJ OA prognosis. The authors recommended a combination of both methods of treatment.
Dalewski et al. (2019) [76]	Randomized controlled clinical trial	90 patients (72 women, 18 men, aged 18–65 years): - splint therapy (30 patients, control group) - splint therapy + nimesulid 100 mg 2 x/day for 14 days (30 patients) - splint therapy + dry needling (30 patients) Occlusal splints were worn only at night-time.	*Endpoint*: three weeks Combination of occlusal splint with nimesulide led to better pain relief compared to the remaining two groups.
Di Rienzo Businco et al. (2004) [77]	Randomized clinical trial	36 patients (19 women, 17 men, aged 34–61 years): - oral diclofenac sodium 50 mg 2 x/day for 14 days (18 patients) - topical diclofenac sodium 16 mg/mL, 10 drops 4 x/day for 14 days, 40 drops corresponded to 1 mL (18 patients).	*Endpoint*: 14 days All patients showed relief from pain after the treatment. The efficacy of both oral administration and multidose topical application of diclofenac sodium was similar.
Ta et al. (2004) [78]	Randomized, double-blindcontrolled clinical trial	68 patients (46 women, 22 men, aged 18–65 years): - celecoxib 100 mg 2 x/day for six weeks (24 patients) - naproxen 500 mg 2 x/day for six weeks (22 patients) - placebo for six weeks (22 patients)	*Endpoint*: six weeks Naproxen appeared to be most effective in TMJ pain reduction, whereas celecoxib was just slightly better than placebo. Naproxen reduced significantly TMJ pain intensity within three weeks of treatment.

TMJ—temporomandibular joint; TMJ OA—temporomandibular joint osteoarthritis; NSAID—nonsteroidal anti-inflammatory drug.

## Data Availability

The data underlying this article are available in the article.
